# Axonal changes in experimental prion diseases recapitulate those following constriction of postganglionic branches of the superior cervical ganglion: a comparison 40 years later

**DOI:** 10.1080/19336896.2019.1595315

**Published:** 2019-04-09

**Authors:** Paweł P. Liberski

**Affiliations:** Laboratory of Electron Microscopy and Neuropathology, Department of Molecular Pathology and Neuropathology, Medical University of Lodz, Lodz, Poland

**Keywords:** Prion, dystrophic neurites, neuroaxonal dystrophy, nerv construction, autophagy, apoptosis

## Abstract

The major neurological feature of prion diseases is a neuronal loss accomplished through either apoptosis or autophagy. In this review, I compared axonal alterations in prion diseases to those described 40 years earlier as a result of nerve ligation. I also demonstrated that autophagic vacuoles and autophagosomes are a major part of dystrophic neurites. Furthermore, I summarized the current status of the autophagy in prion diseases and hypothesize, that spongiform change may originate from the autophagic vacuoles. This conclusion should be supported by other methods, in particular laser confocal microscopy.

We observed neuronal autophagic vacuoles in different stages of formation, and our interpretation of the ‘maturity’ of their formation may or may not equate to actual developmental stages. Initially, a part of the neuronal cytoplasm was sequestrated within double or multiple membranes (phagophores) and often exhibited increased electron-density. The intracytoplasmic membranes formed labyrinth-like structures that suggest a multiplication of those membranes. The autophagic vacuoles then expand and eventually, a vast area of the cytoplasm was transformed into a merging mass of autophagic vacuoles. Margaret R. Matthews published a long treatise in the *Philosophical Transactions of the Royal Society of London* in which she had described in great detail the ultrastructure of postganglionic branches of the superior cervical ganglion in the rat following ligation of them. The earliest changes observed by Matthews between 6 h to 2 days in the proximal stump were distensions of proximal axons. Analogously, in our models, an increased number of ‘regular’ (round) and ‘irregular’ MVB and some autophagic vacuoles were observed collectively, both processes were similar.

## Introduction

Prion diseases are a group of noninflammatory transmissible diseases of the central nervous system (CNS) of both humans and animals. In humans, they comprise kuru, Creutzfeldt-Jakob disease (CJD; sporadic, familial, iatrogenic and variant); Gerstmann-Sträussler-Scheinker (GSS) disease and fatal familial insomnia. In animals, they include scrapie, bovine spongiform encephalopathy (BSE), chronic wasting disease (CWD) in captive and wild cervids and transmissible mink encephalopathy (TME) in ranch-reared mink. Recently a prion disease in dromedary camel was described [1]. They are caused by unusual infectious agent designated prion, from *proteinaceous infectious particles*, widely believed to be composed exclusively of an abnormal isoform (PrP^Sc^) of a normal cellular protein (PrP^c^). How PrP^c^ converts to PrP^Sc^, whether a cofactor is needed and how the aggregate of PrP^Sc^ becomes infectious has not been well envisaged [2].

**Figure 13.**
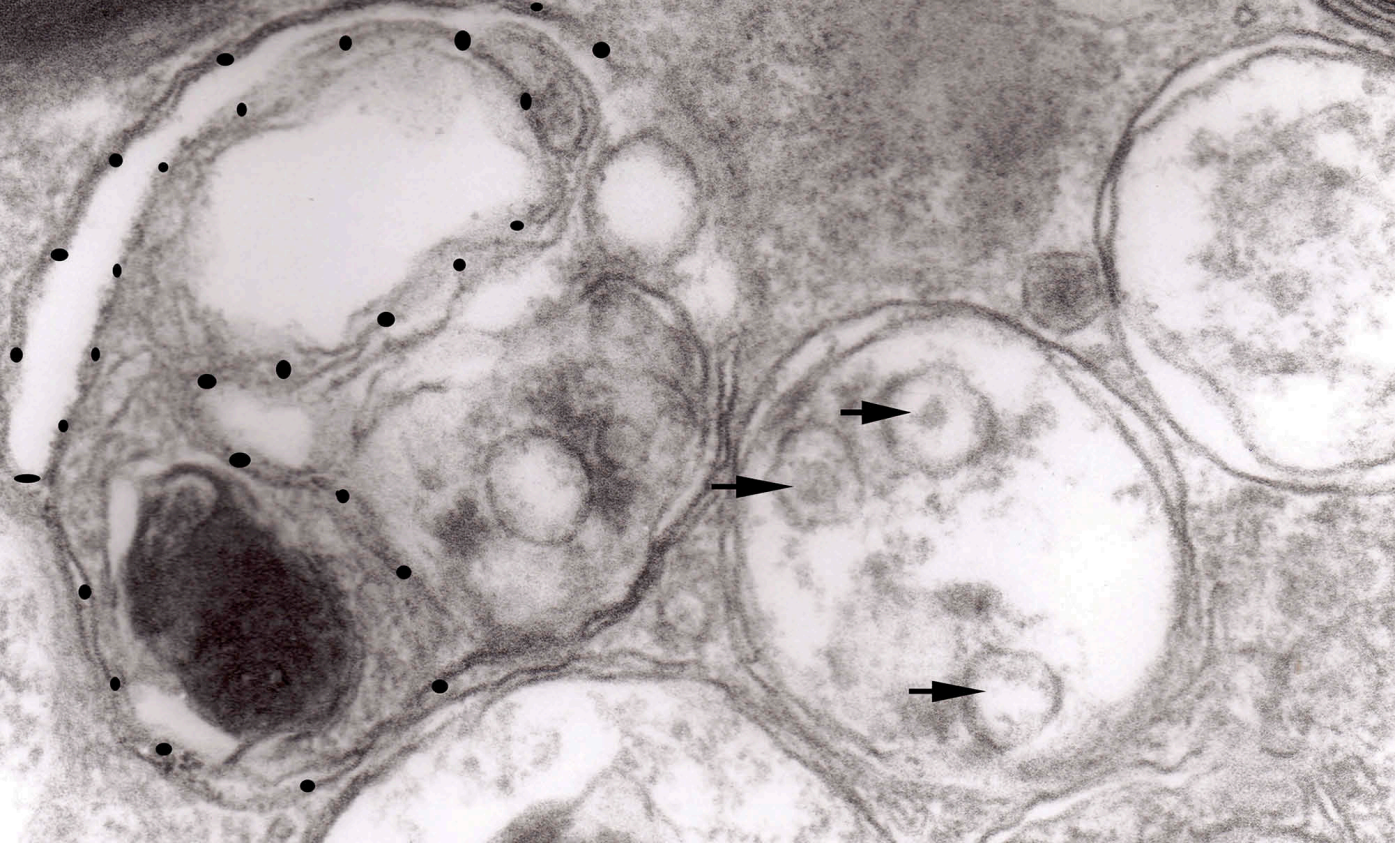
Three autophagic vacuoles in different stages of formation. The left one presents two adjacent membranes marked with black ovals; those membranes form a loop. The middle autophagic vacuole contains three electron-lucent vesicles (additional autophagic vacuoles ?); original magnification, x 33,000.

Prion diseases are neurodegenerative disorders – i.e. they are not inflammatory disorders [3]. It has been recently suggested that other neurodegenerative disorders, i.e. Alzheimer disease, Parkinson’s disease, frontotemporal dementias and many more are in reality prion-like diseases caused by misfolding proteins [4,5]. It seems also that ‘infectious’ proteins may be transferred horizontally as ‘regular’ prions [6,7]. The neuropathological alterations of prion diseases consist of spongiform change of the neuropil or in neurons, the latter more characteristic for animal diseases, astrocytosis, a microglial response and, in some but not all diseases, amyloid plaques. The major neurological feature is a neuronal loss accomplished through either apoptosis or autophagy. In this review, we shall compare axonal alterations in prion diseases to those described some 40 years earlier as a result of nerve ligation. We are aware that electron microscopy is subject to bias; however, we compared structures that are easy to pick up against the background. Thus, we believe that we noticed every one of them in all model. We shall also demonstrate that autophagic vacuoles and autophagosomes are a major part of dystrophic neurites. Furthermore, we shall summarize the current status of the autophagy in prion diseases and hypothesize, that spongiform change may originate from the autophagic vacuoles. This conclusion should be supported by other methods, in particular laser confocal microscopy.

## Neuronal cell death in TSEs

As in many neurodegenerative diseases caused by the accumulation of ‘toxic’ misfolded proteins [8,9] prion diseases die via programmed cell death of which only the apoptotic process is relatively well understood. According to the recommendations of the Nomenclature Committee on Cell Death [10–12], three major types of programmed cell death (PCD) can be discriminated.

The first type (apoptosis) is characterized by electron microscopy by specific alterations – cell shrinkage, condensation of chromatin and, eventually, the formation of so-called ‘apoptotic bodies’ that are actively phagocytosed by macrophages. Apoptosis is regulated by a highly conservative network of molecules consisting of Bcl-2 family, caspases, and many others, but some evidence suggests that apoptosis without caspases activation may also occur [13].

The second type – involving macroautophagy (called ‘autophagy’ through this text) – is characterized by the presence of numerous autophagosomes that subsequently fuse with lysosomes to form autolysosomes. The molecular mechanism differs from that of apoptosis and consists of a complex interplay of numerous proteins including the mTor (mammalian target of rapamycin) kinase.

The third type is similar to the second type, except for the negligible or absent involvement of lysosomes. Electron microscopically, type 3 cell death is characterized by swelling of intracellular organelles resulting in the formation of large empty spaces within the cytoplasm. Of interest, TSEs are characterized by ‘spongiform vacuole’ formation within neuronal elements. While the latter have never been linked to the type 3 PCD, the ultrastructural resemblance of ‘large empty spaces’ to ‘spongiform vacuoles’ appears to be worthy of consideration (see below).

The Nomenclature Committee on Cell Death [10] also recognizes ‘mitotic catastrophe’, ‘anoikis’, ‘cornification’, ‘Wallerian degeneration’ and ‘excitotoxicity’: these will not be discussed here. It should be stressed, however, that the major categories of cell death have been defined based mostly on *in vitro* observations, and the strict usage of such formulated criteria for tissues *in vivo* may not be entirely appropriate.

As an evolutionarily ancient cellular response to intra- and extracellular noxious stimuli, autophagy may precede or co-exist with apoptosis, and the process may be induced by apoptotic stimuli. Furthermore, the level of autophagy may define the sensitivity of a given neuronal population to apoptotic stimuli, which may underlie the phenomenon of ‘selective neuronal vulnerability’. Thus, autophagy and apoptosis are often interwoven.

Cellular autophagy is a physiological degradation process employed, like apoptosis, in embryonic growth and development, cellular remodelling and the biogenesis of some subcellular organelles ‒ viz. multilamellar bodies [14,15]. A portion of the cytoplasm is engulfed by a semi-circular membrane which closes to create a double membrane vesicle – an autophagosome [16]. Autophagosomes fuse with lysosomes and deliver its cargo to them to form autolysosomes and, as in apoptosis, only excessive or misdirected autophagy causes a pathological process basically regarded as protective against aggregation of misfolded proteins, including PrP^Sc^. Autophagy is highly enhanced in many brain amyloidoses or conformational disorders, Alzheimer’s disease, Parkinson’s disease [17] and Huntington’s disease, in which the signal for autophagy is Huntington [18]. Autophagy is largely regarded as a protective mechanism helping in the removal by organelles of misfolded proteins [19,20].

## Neuronal autophagy in prion diseases: ultrastructural observations

Data on autophagy in prion diseases and in yeast prions are meagre [21–26]. Our initial strategy using the hamster-adapted 263K or 22C-H strains of scrapie [26–30] was subsequently broadened by exploration of human brain biopsies from patients with sporadic CJD, variant CJD, and FFI [31,32]. Experimentally infected animal prion disease models are widely used because of their relatively short incubation periods that, for mice, range from 16 to 18 weeks, and for hamsters from 9 to 10 weeks for the 263K scrapie strain and 24–26 weeks for the 22C-H scrapie strain.

For human TSE strains, mice were inoculated either with the Fujisaki (Fukuoka 1) strain of GSS [33,34], and hamsters were inoculated with either the Echigo-1 strain of CJD or the 263K strain of scrapie [28,35]. The Echigo-1 strain of CJD was isolated by Mori and colleagues [35] from a case of 33-year-old female with a panencephalopathic type of GSS [36]. The inoculum was originally prepared from brain tissue and was passaged through guinea pigs with incubation periods (IP) of 728 days at primary and approximately 400 days at subsequent passages. From an animal that exhibited hyperactivity and an excessive response to external stimuli, a substrain was isolated with substantially reduced IP (254 days). This strain was re-isolated in hamsters with IP of 141 days at the third passage. Control animals were sham-inoculated with saline. The Fujisaki strain of GSS was isolated from a GSS case by Tateishi et al. [37].

## Formation of autophagic vacuoles in prion-affected brains

Autophagic vacuoles developed not only in neuronal perikaryal but also in neuronal processes eventually replacing the whole cross-section of affected neuritis. In a few specimens, we found round electron-dense structures that we identified as aggresomes [38]. In general, there was little qualitative difference between hamsters infected with either the 263K strain of scrapie or the 22L-H strain, although hamsters inoculated with the 263K strain showed a more robust pathology.

Autophagic vacuoles are areas of the cytoplasm sequestrated within double or multiple membranes (phagophores) of unknown origin; the endoplasmic reticulum may be a possible source. Sequestrated piece of the cytoplasm within membranes contains ribosomes, small secondary vacuoles, and occasional mitochondria. Some vacuoles present a homogeneously dense appearance.

We observed neuronal autophagic vacuoles in different stages of formation in the same specimens, and our interpretation of the ‘maturity’ of their formation may or may not equate to actual developmental stages. Initially, a part of the neuronal cytoplasm was sequestrated within double or multiple membranes (phagophores) and often exhibited increased electron-density. The intracytoplasmic membranes formed labyrinth-like structures that suggest a multiplication of those membranes. The autophagic vacuoles then expand and eventually, a vast area of the cytoplasm was transformed into a merging mass of autophagic vacuoles.

## Autophagy and neuritic degeneration

Our interest in neuritic changes in prion disease is a long-lasting one [39–41]. The interpretation of neuritic changes in prion diseases has changed, however. In a seminal Lampert [42] papers, definitions of ‘dystrophic’, ‘regenerative’ and ‘degenerative’ neurites were established based on transmission electron microscopy and accumulations of altered mitochondria and heterogeneous dense bodies were regarded as an ultrastructural correlate dystrophic neurites. With the passage of time, it appeared that distinction between those three classes of neurites are not that clear cut [43] and we used the term ‘dystrophic neurites’ as an ‘umbrella term’ to cover all classes of altered neuronal branches. Furthermore, in the quantitative study, we observed that alterations in the number of dystrophic neurites during the incubation period involved only those neurones containing more than one dense body [41]. Recently a change in the interpretation of the nature of those accumulations has occurred as following a renewed interest in autophagy. Nixon et al. [44,45] interpreted many of those dense-bodies as small autophagic vacuoles.

Of interest, in 1973 Margaret R. Matthews published a long treatise in the *Philosophical Transactions of the Royal Society of London* [46] in which she had described in great detail, the ultrastructure of postganglionic branches of the superior cervical ganglion in the rat following ligation of them. This study was based on enormous number, in terms of electron microscopy, of 48 rats that underwent either cutting or constriction of external carotid nerve/or internal carotid nerve. Those animals were left to survive for until 143 days and electron microscopic alterations were observed and categorized.

In the following paragraph, I decided to re-arrange our electron microscopic data on neuroaxonal dystrophy in prion diseases as to follow the findings in the Matthews review and to compare those two interpretations.

**A putative sequence of changes in neurites in hamster brains infected with the Echigo-1 and the 263K strains of CJD and scrapie, respectively, as deduced against alterations published for sympathetic nerves constrictions** [46].

**Figure 1.**
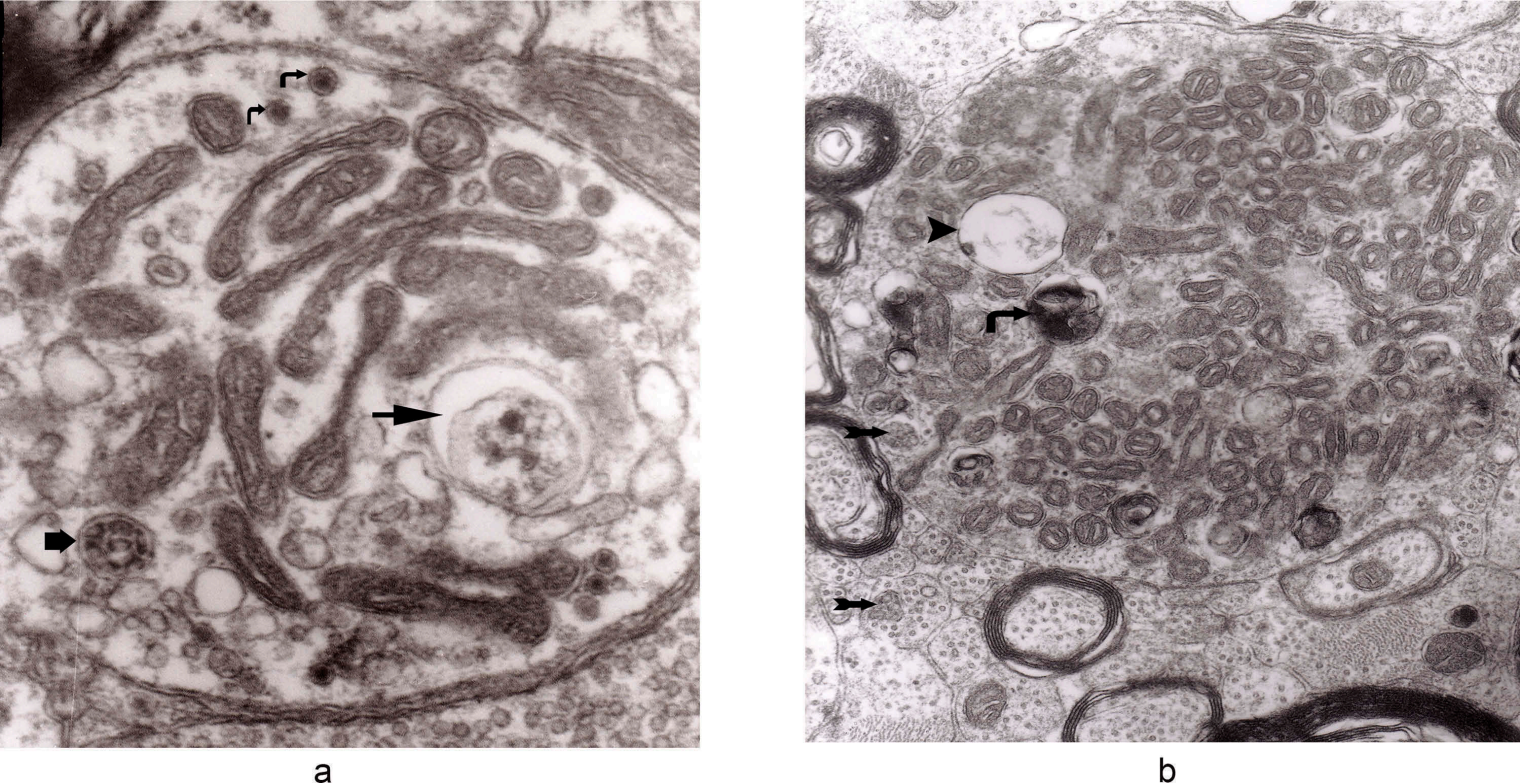
(a, b) An axon in an early phase of degeneration. There are numerous slender mitochondria, a multivesicular body (short arrow) and a small autophagic vacuole (arrowhead). Numerous lucent vesicles and dense-core vesicles (bent arrows) are observed. Original magnification, x 8300.

**Figure 2.**
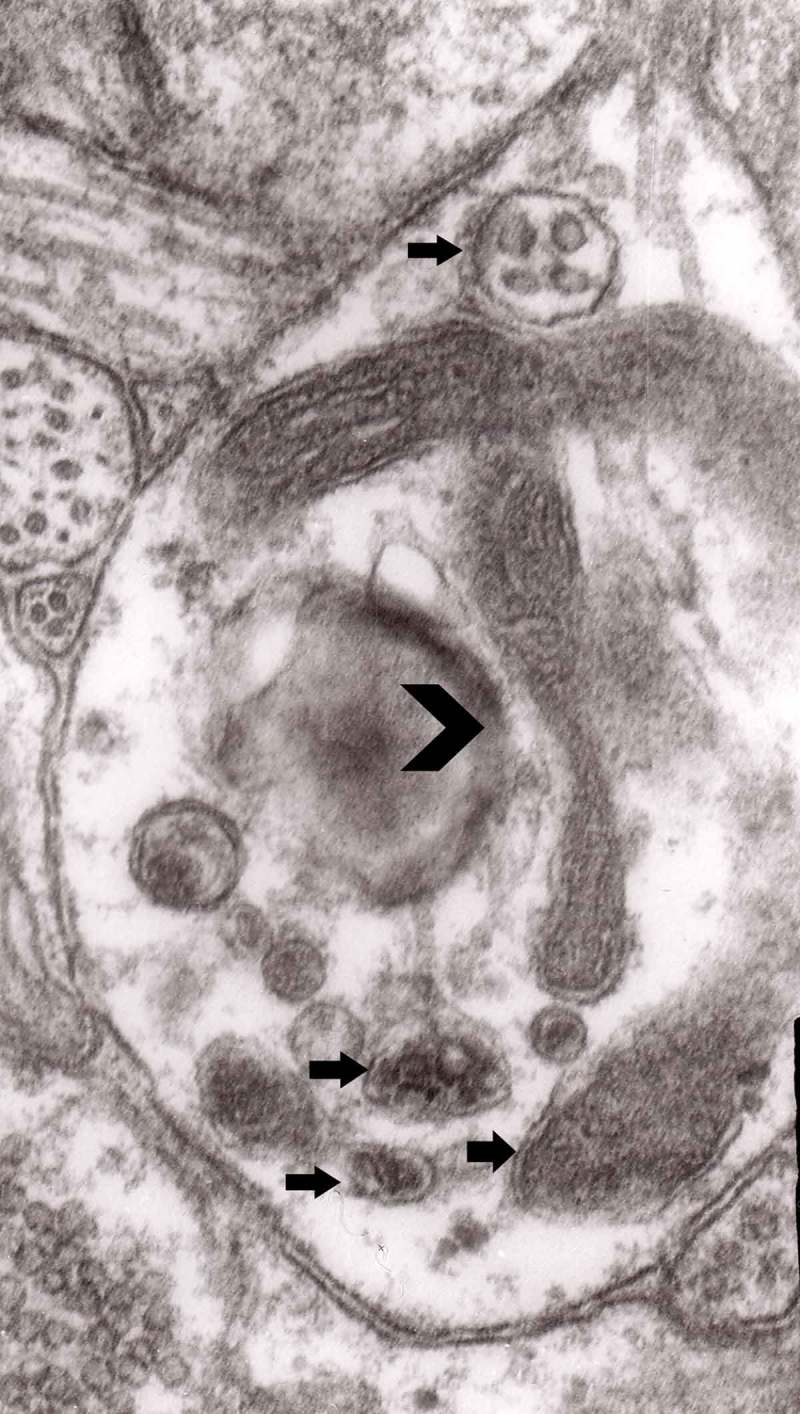
Degenerating axon accumulating some elongated mitochondria, ‘regular’ and ‘irregular’ MVB (arrows) and early autophagic vacuole (arrowhead pointing toward the autophagosome membrane); original magnification, x 8300.

The earliest changes observed by Matthews between 6 h to 2 days in the proximal stump were distensions of proximal axons. Those distensions were relatively large encompassing up to 0.4 μm of the axons and were already filled with packed organelles comprising mitochondria, vesicles and short tubular profiles (Figure 1(a,b)). By the 12th and 13th hours in the Matthews experiment, mitochondria appeared clustered and these changes were also readily observed in our models (Figure 1b). Multivesicular bodies (MVB), dense cytoplasmic bodies (DCB) and clumps of electron-dense vesicles were also seen. Analogously, in our models, an increased number of ‘regular’ (round) and ‘irregular’ MVB and some autophagic vacuoles were observed (Figure 2).

**Figure 3.**
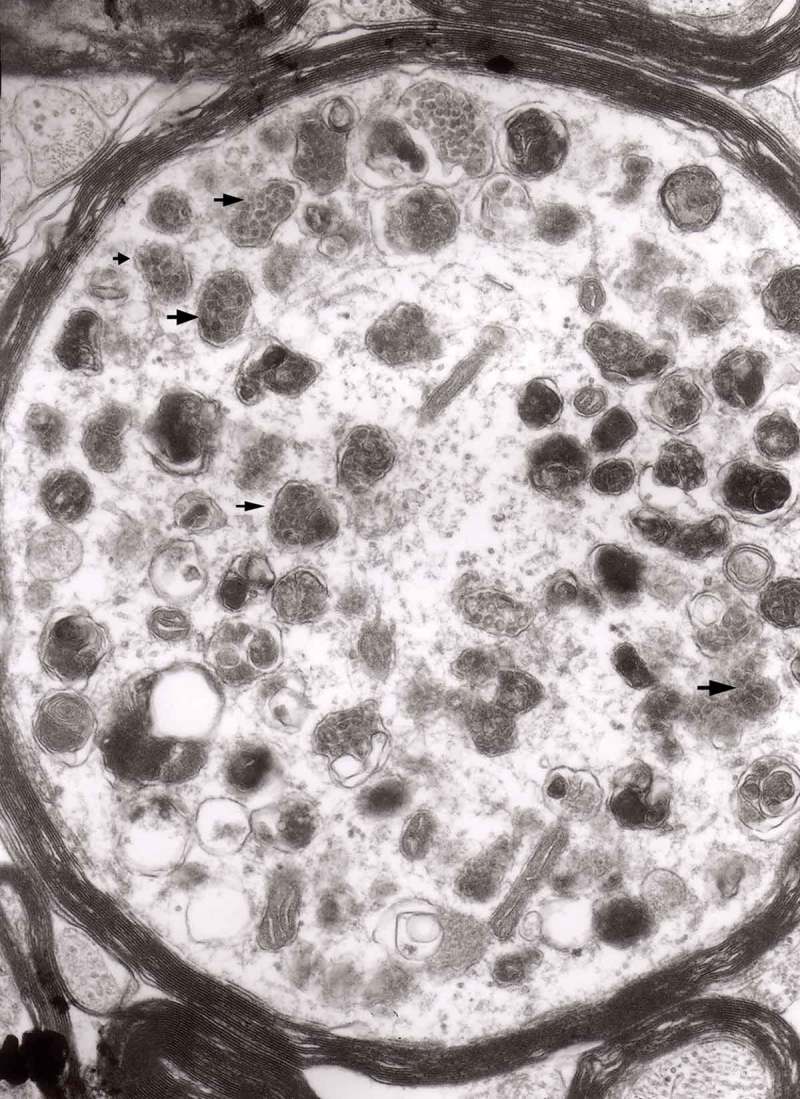
Dilated myelinated axons filled with a large number of MVB (arrows). This electron-micrograph is analogous to Figure 16. of Matthews [46]. Note that some MVB take part in a formation of autophagic vacuoles and Figure 5, original magnification, x 33 000.

**Figure 4.**
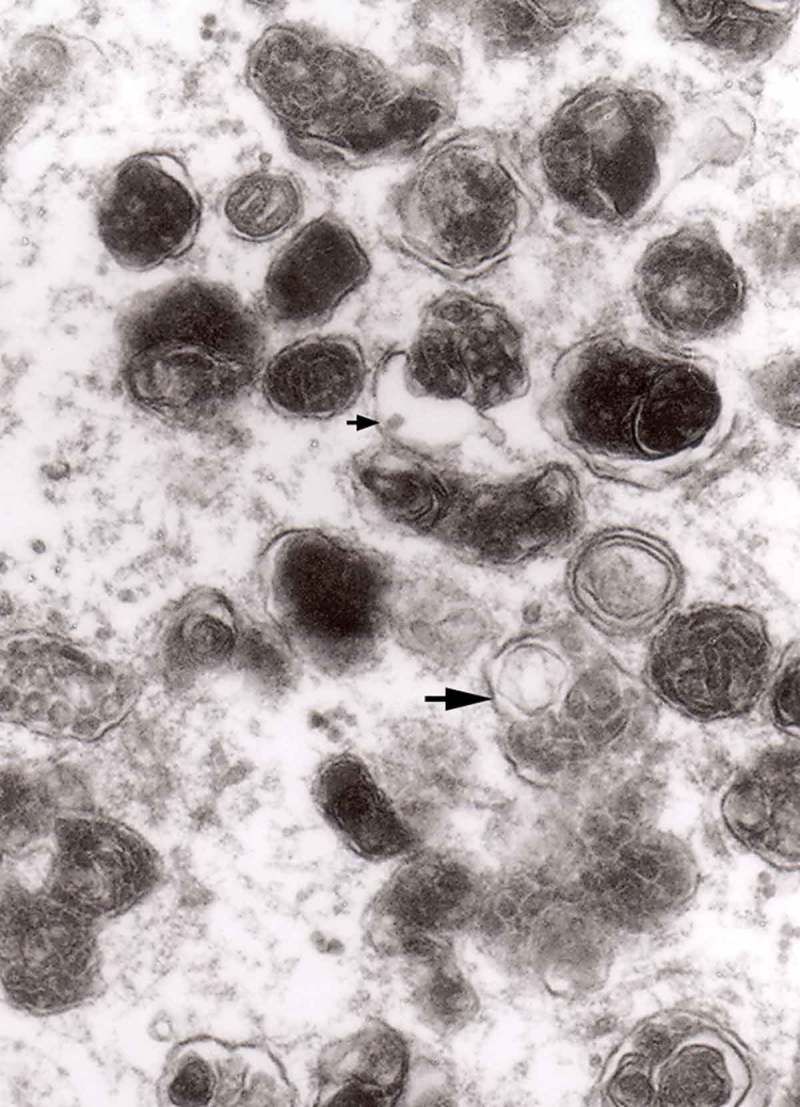
Higher magnification of part of dystrophic neurite depicted in Figure 3. Note that some ‘irregular’ MVB take part in autophagic vacuole formation.

**Figure 5.**
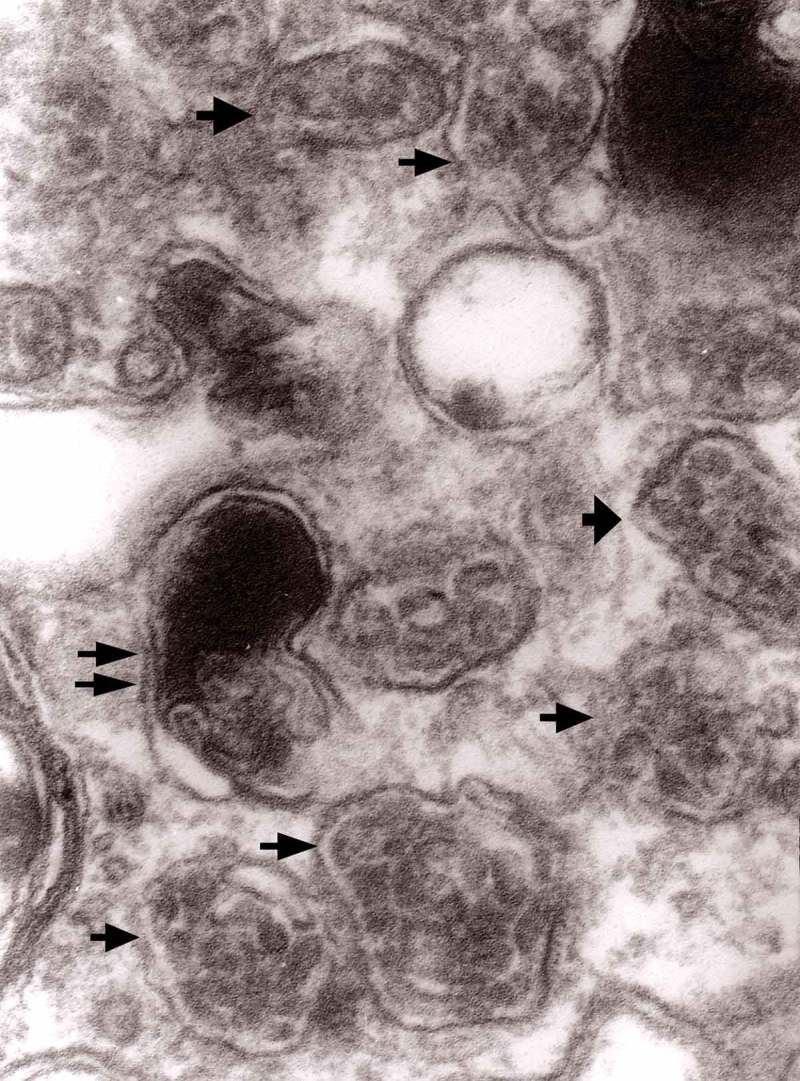
A large magnification of numerous ‘irregular’ MVB (arrows). This electron micrograph corresponds to Figure 19. of Matthews. Note that MVB marked with double arrows presents a dark part, probably an autophagic vacuole. Original magnification, x 20 000.

**Figure 6.**
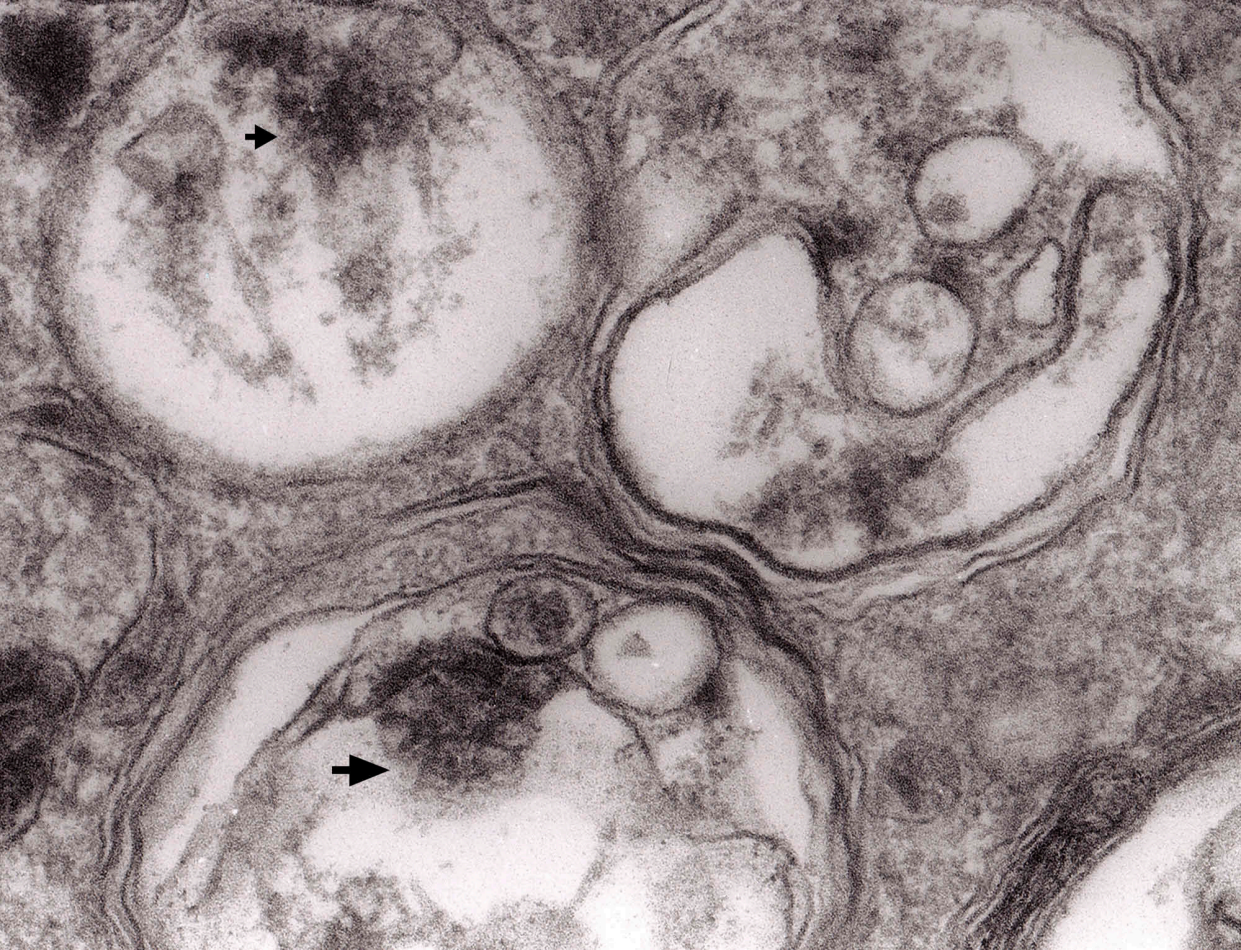
A fragment of a dystrophic axon containing three autophagic vacuoles with content (arrows) of ‘different stages of darkening and degradation’, original magnification, x 33,000. This figure corresponds to Matthews Figure 21.

**Figure 7.**
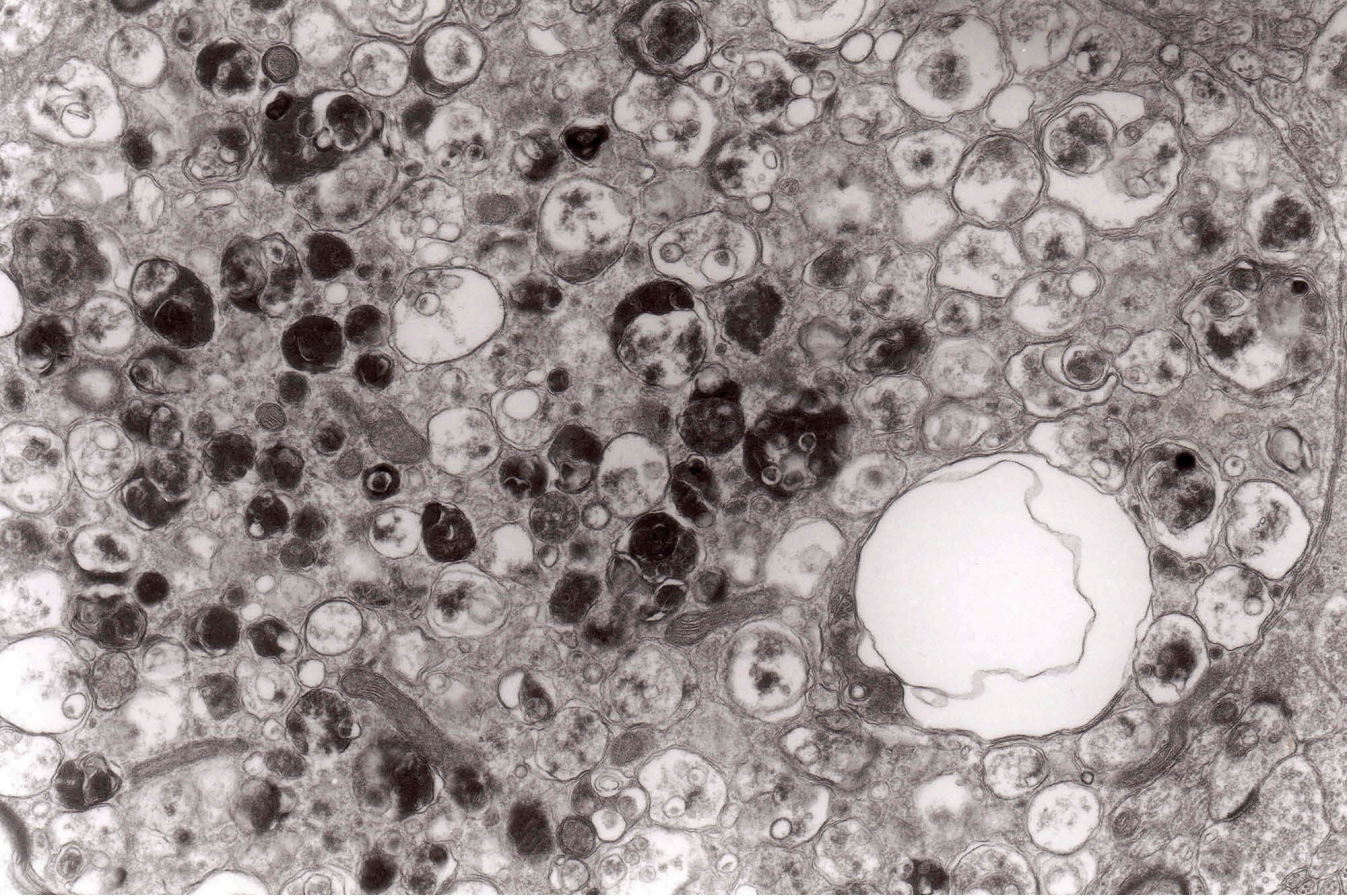
A fragment of a dystrophic axon containing numerous dense bodies and ‘forming and formed’ autophagic vacuoles; original magnification, 6600. This figure corresponds to Matthews Fig. 22. The details are shown in Figures 8 and 9.

**Figure 8.**
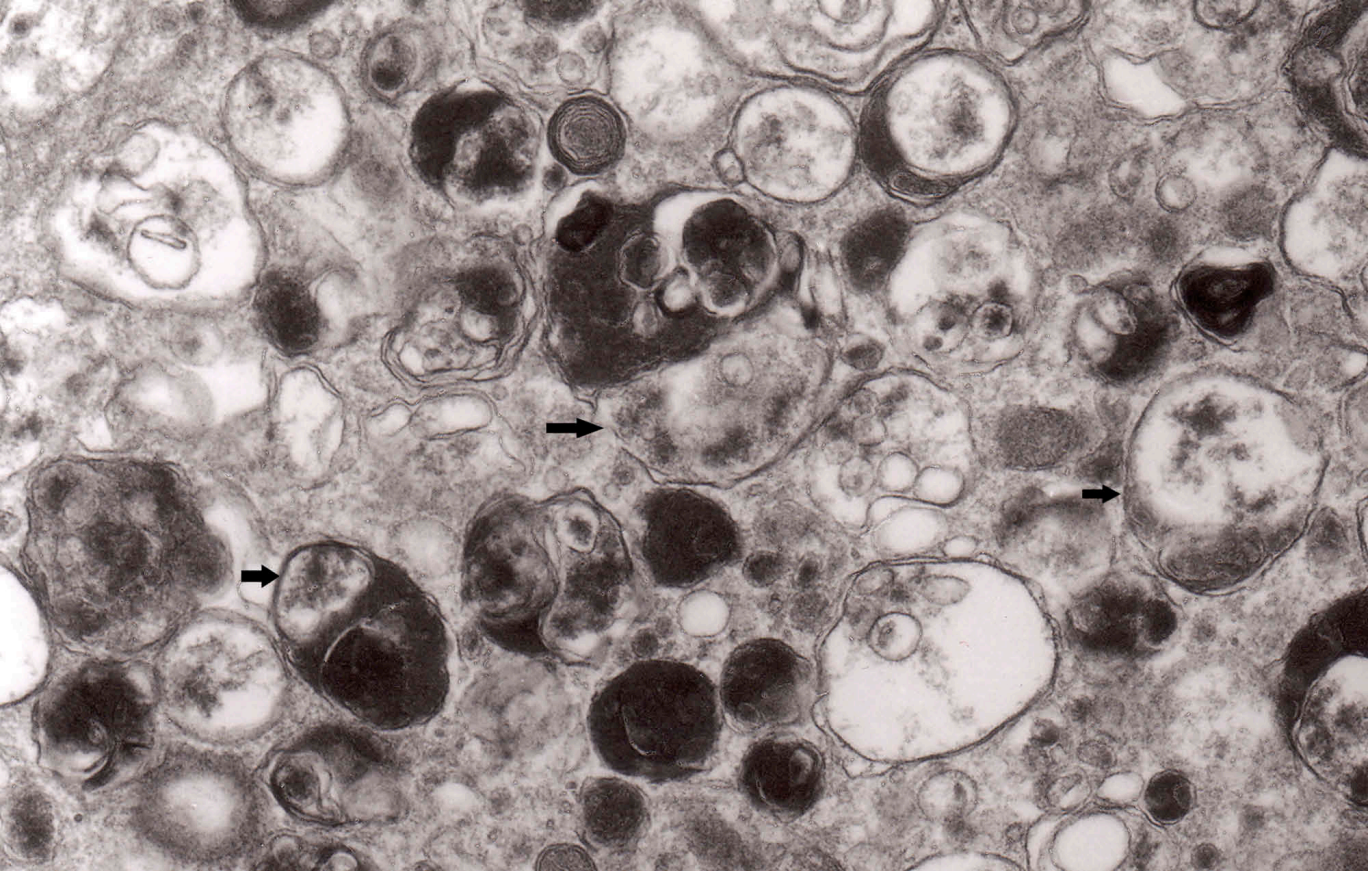
A fragment of Figure 7. Note autophagic vacuoles (arrows) with partially electron-dense content; original magnification, x 6600.

**Figure 9.**
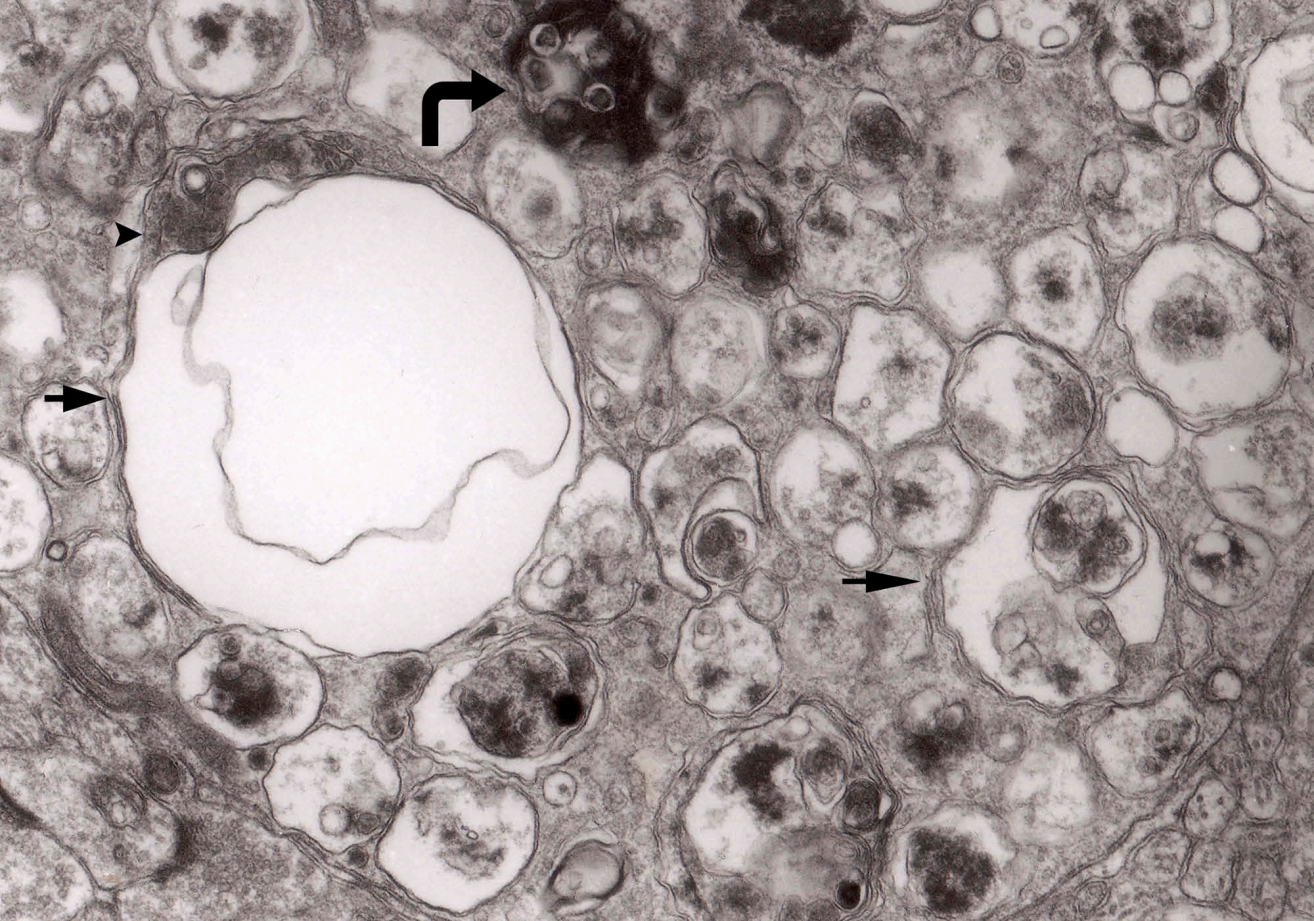
Another fragment of Figure 7. Note two larger autophagic vacuoles (arrows) and a complex dense body. Note also, that larger vacuole of those two contains electron-dense fragment, this corresponds to partially digested content.

From 12 and 13 hours onward, the clusters of mitochondria were seen in Matthews experiment (Matthews, Figures 2–5; mine, Figures 1–2) followed by increased number of DCB, MVB and autophagic vacuoles. By 38 h following constriction, Matthews observed typical dystrophic neurites filled with ‘masses of DCB’ and a large number of autophagic vacuoles. Our data closely followed the Matthews description (Figures 3–9). Complex ‘irregular’ MVB taking part in the formation of autophagic vacuoles were visible (Figures 4–5). In the latter situation, the MVB form a ‘cap’ sitting on a membrane enveloping electron-lucent vacuole. Numerous autophagic vacuoles also exhibit, as Matthews described, different stages of darkening and degradation (Figures 6–9).

**Figure 16.**
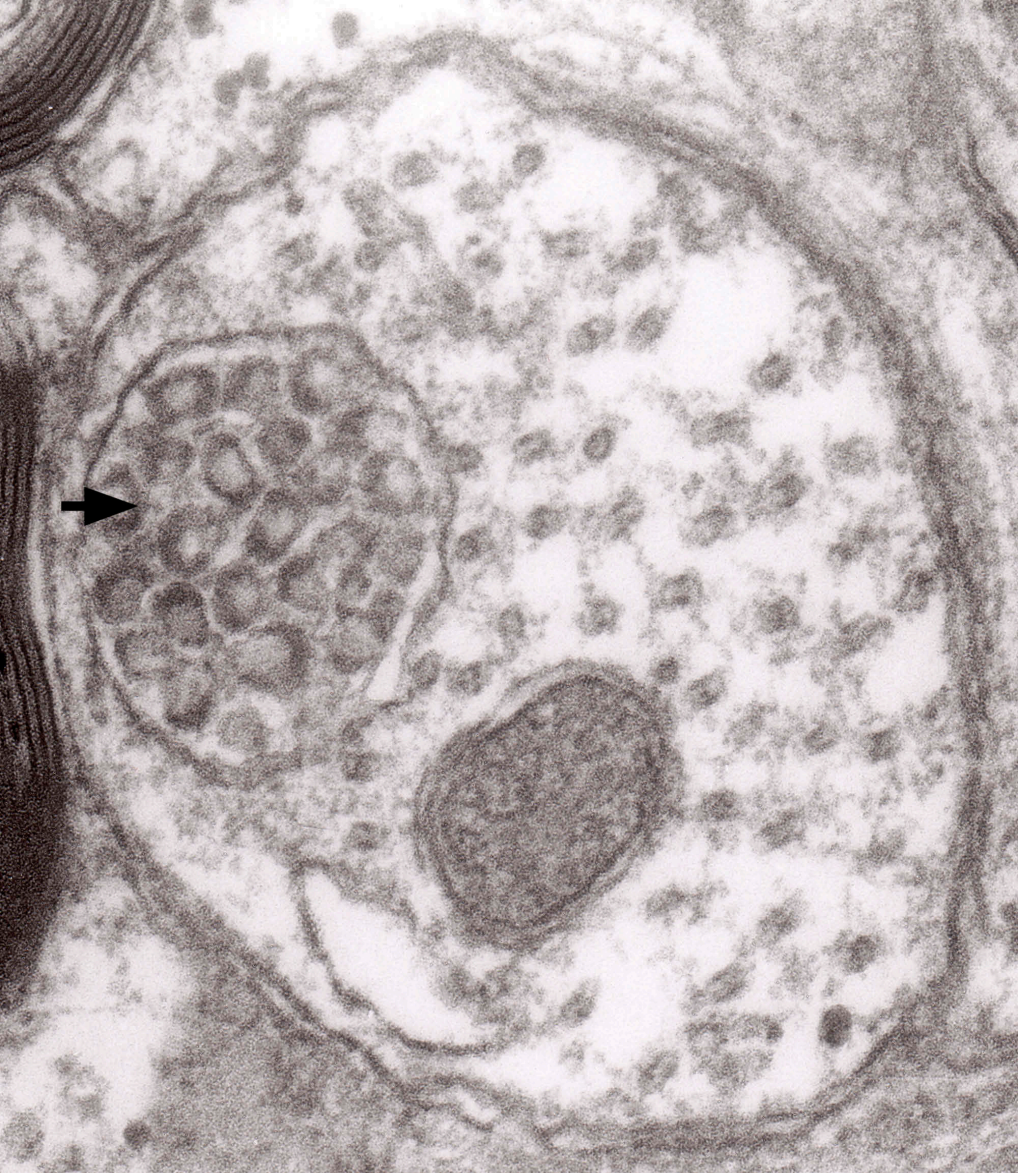
A typical round ‘regular’ MVB in unmyelinated nerve process; original magnification, x 16,000.

**Figure 19.**
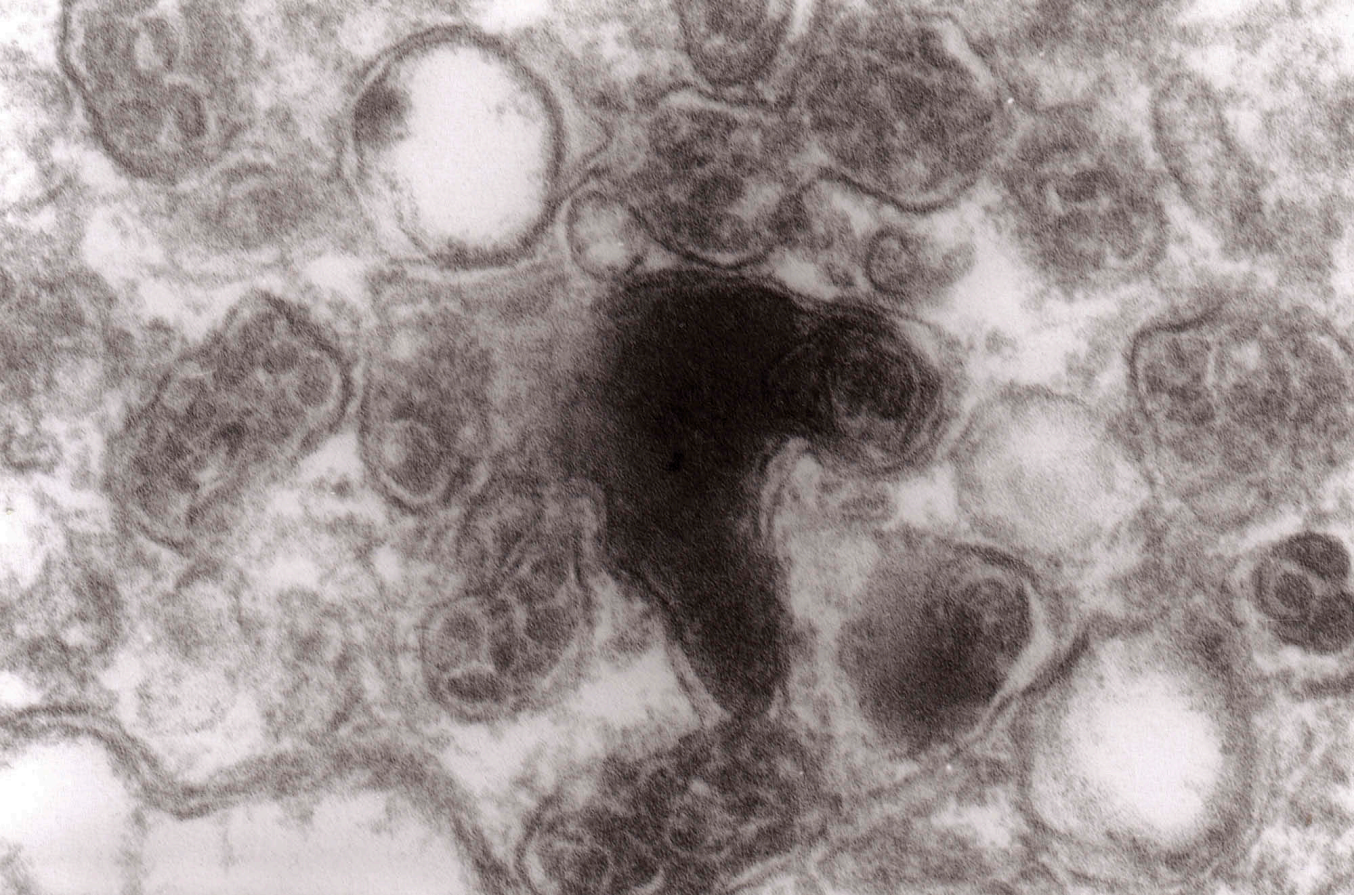
Accumulation of numerous MVBs of irregular sizes and partially flattened; original magnification, x 33,000.

**Figure 21.**
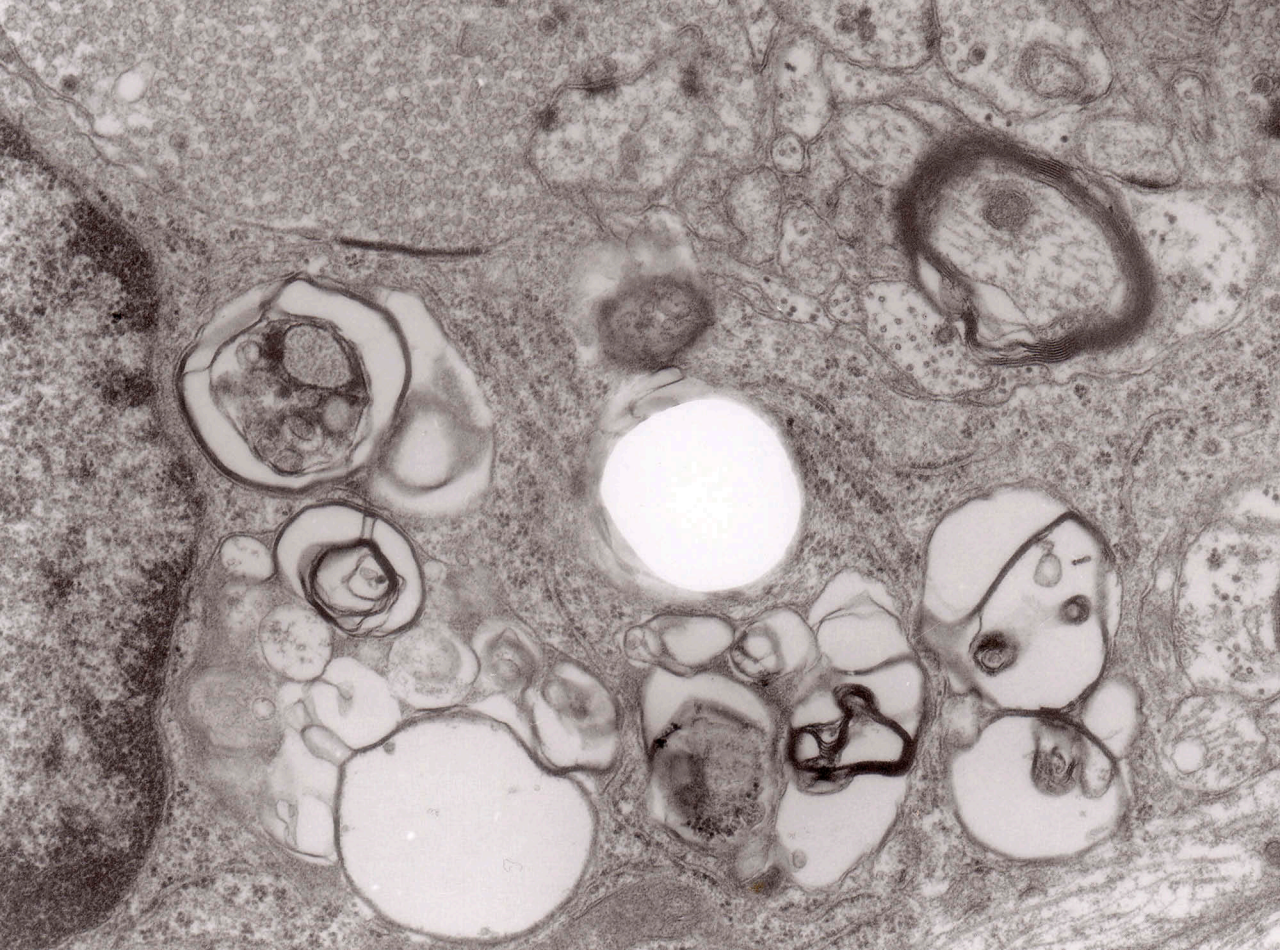
Another fragment of cytoplasm of the macrophage filled with numerous lysosomes and autophagic vacuoles, original magnification, x 16 000.

**Figure 10.**
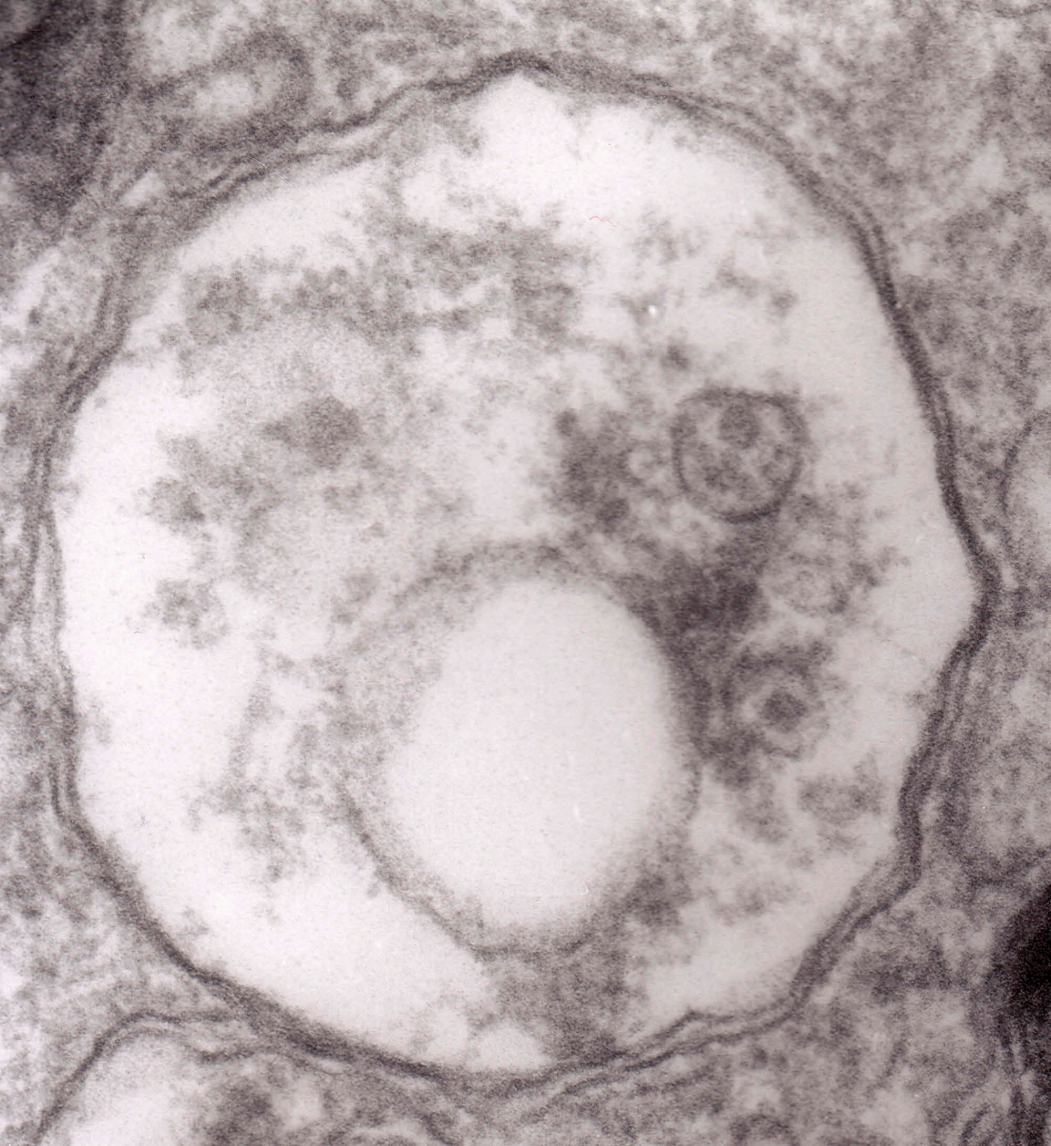
An enlarged fragment of dystrophic neurite showing an autophagic vacuole lined with double membrane. Original magnification, x 33,000.

**Figure 11.**
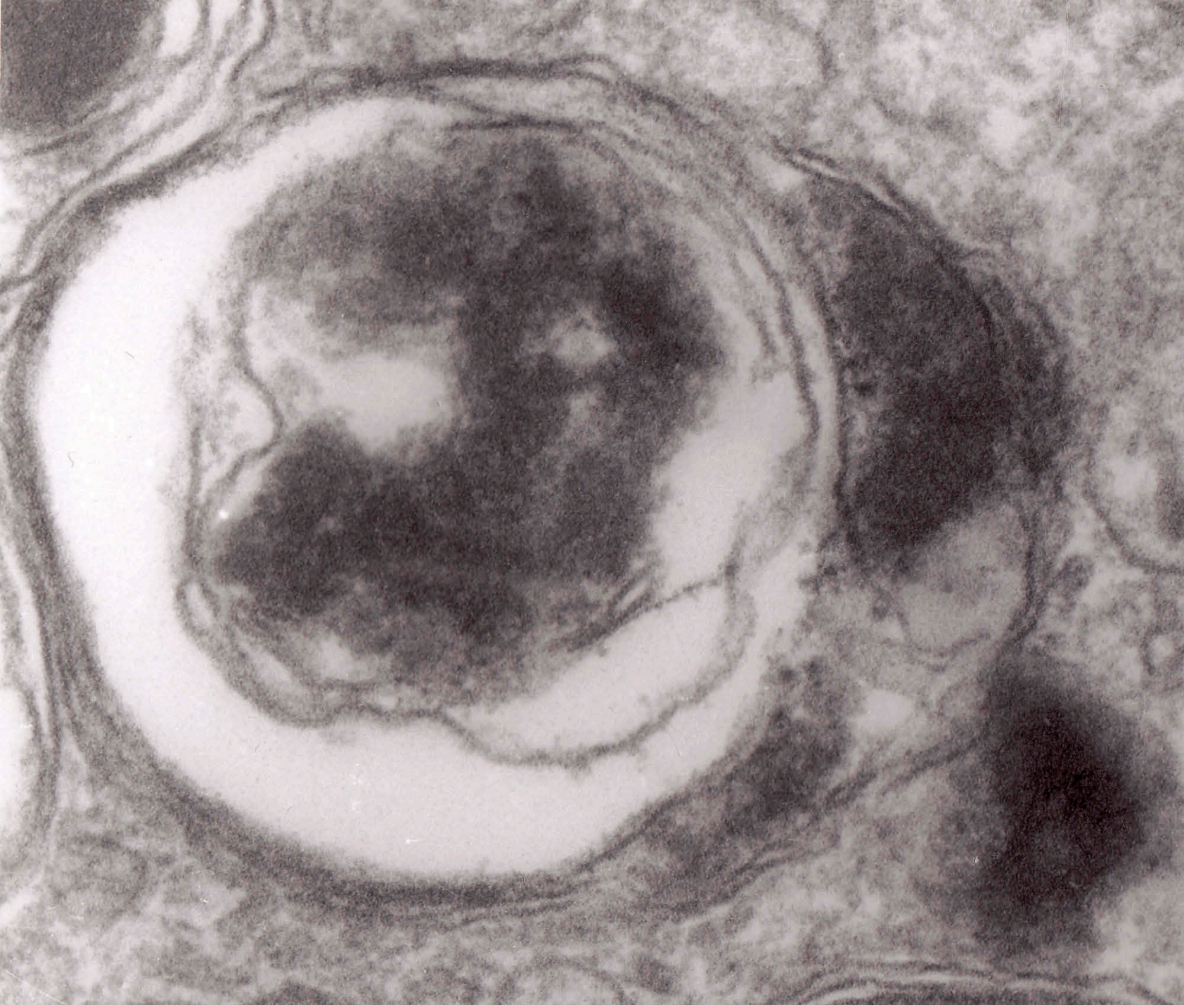
An enlarged fragment of dystrophic neurite showing a complex autophagic vacuole with a darkened content. Original magnification, x 33,000.

**Figure 12.**
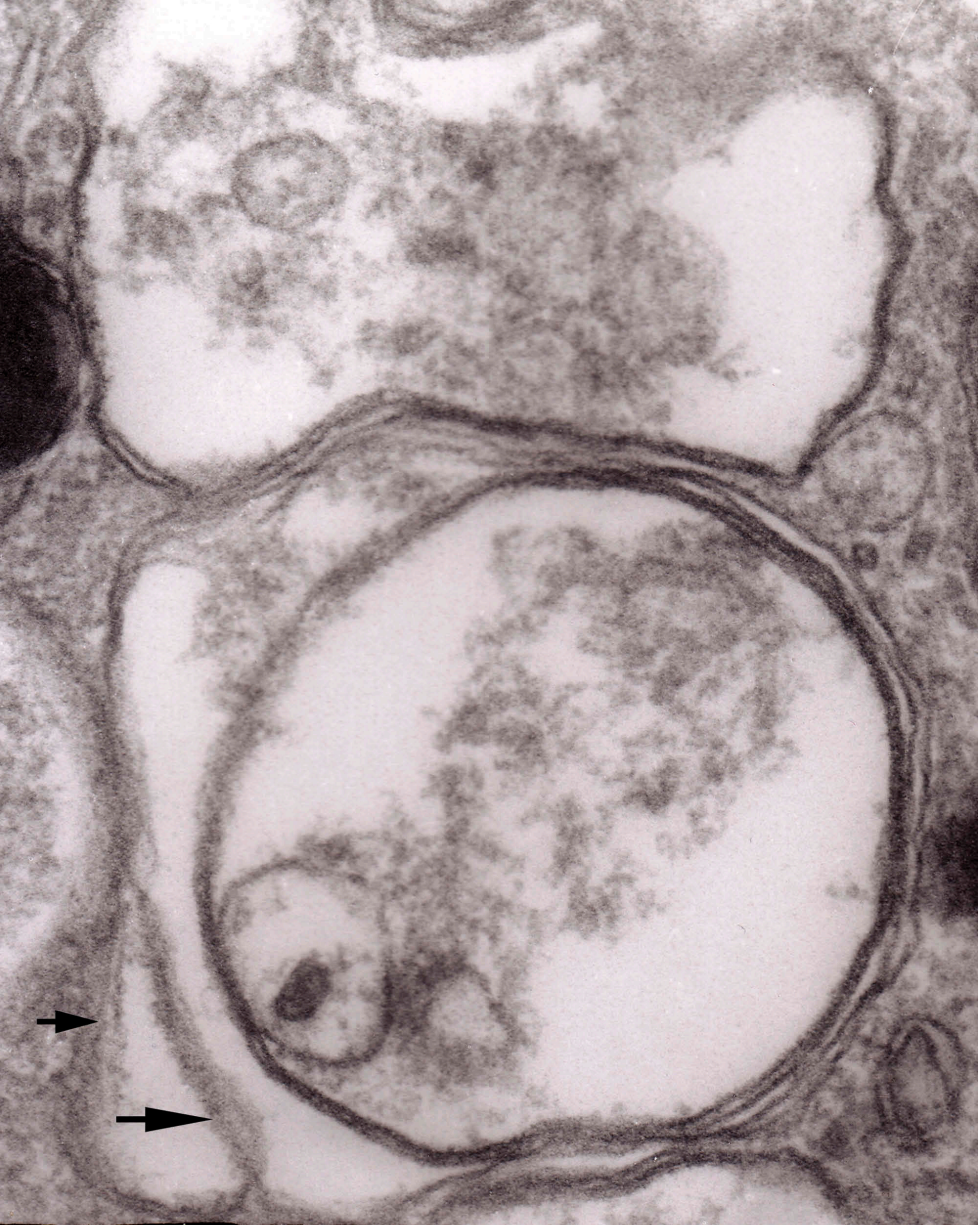
An enlarged fragment of dystrophic neurite showing a complex autophagic vacuole with the formed loop (two arrows) penetrating the adjacent cytoplasm. Original magnification, x 33,000.

**Figure 14.**
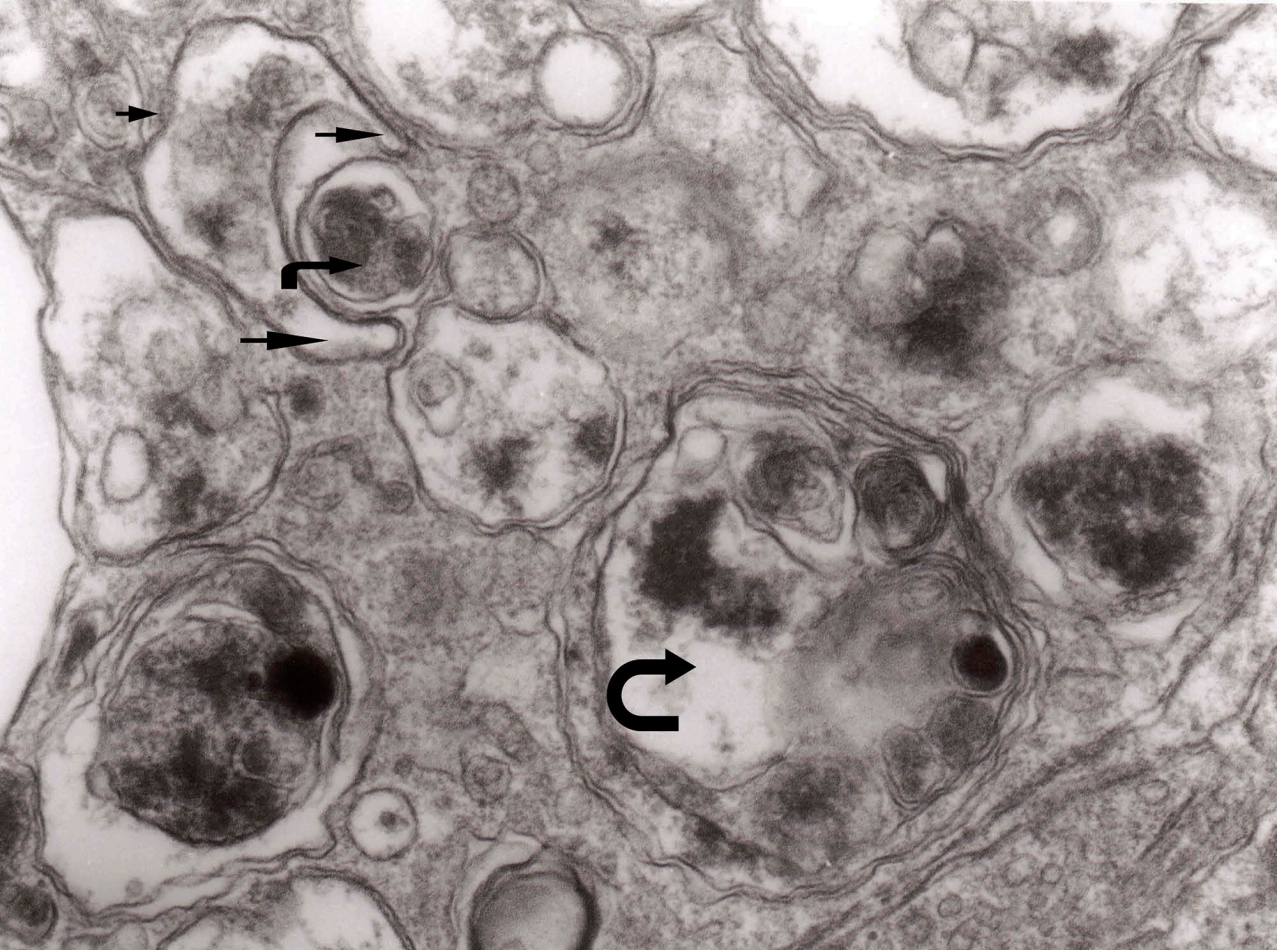
Several autophagic vacuoles. Note the one marked with arrows – it demonstrates elongated protrusions in a process of encircling another vacuole (bent arrow). A complex autophagic vacuole is visible in the vicinity (semicircular arrow); original magnification, x 33,000.

**Figure 15.**
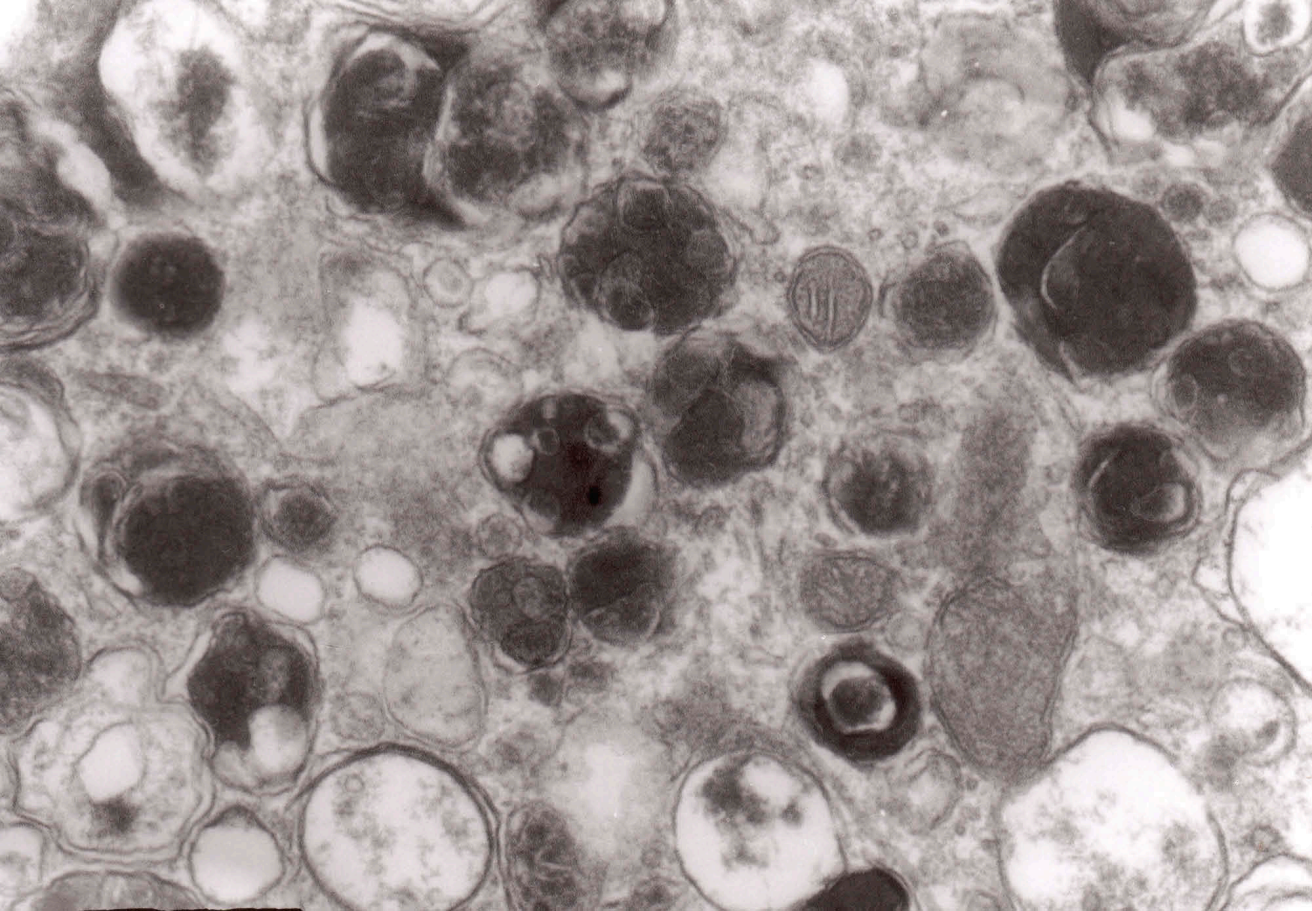
Accumulation of dense bodies – the similarities or even identity with autophagic vacuoles is noticeable; original magnification, x 20,000.

The formation of autophagic vacuoles may be followed in our material and closely corresponds to those depicted by Matthews. It must be stressed, however, that we compared chronic experimental prion diseases with a model of acute nerve ligation. On the basis of our findings, we suggest that the changes observed on electron microscopy in chronic experimental prion diseases represent numerous episodes of the acute changes observed following nerve ligation, seen in cross section. Autophagic vacuoles are enclosed by paired membranes (Figure 10; corresponding to Matthews Figures 21–22). These membranes proliferate, form loops or ‘flattened sacs’ and encircle the fragment of the cytoplasm to form a complex autophagic vacuole composed of several chambers (Figure 11). Such loops in the process of penetrating the cytoplasm are occasionally discernible (Figures 12–14); of note, such a loop is somehow reminiscent of a loop of inner mesaxon during myelin formation. Cytoplasmic dense bodies are frequent and their similarity to autophagic vacuoles was stressed by Matthews (Figure 15).

**Figure 17.**
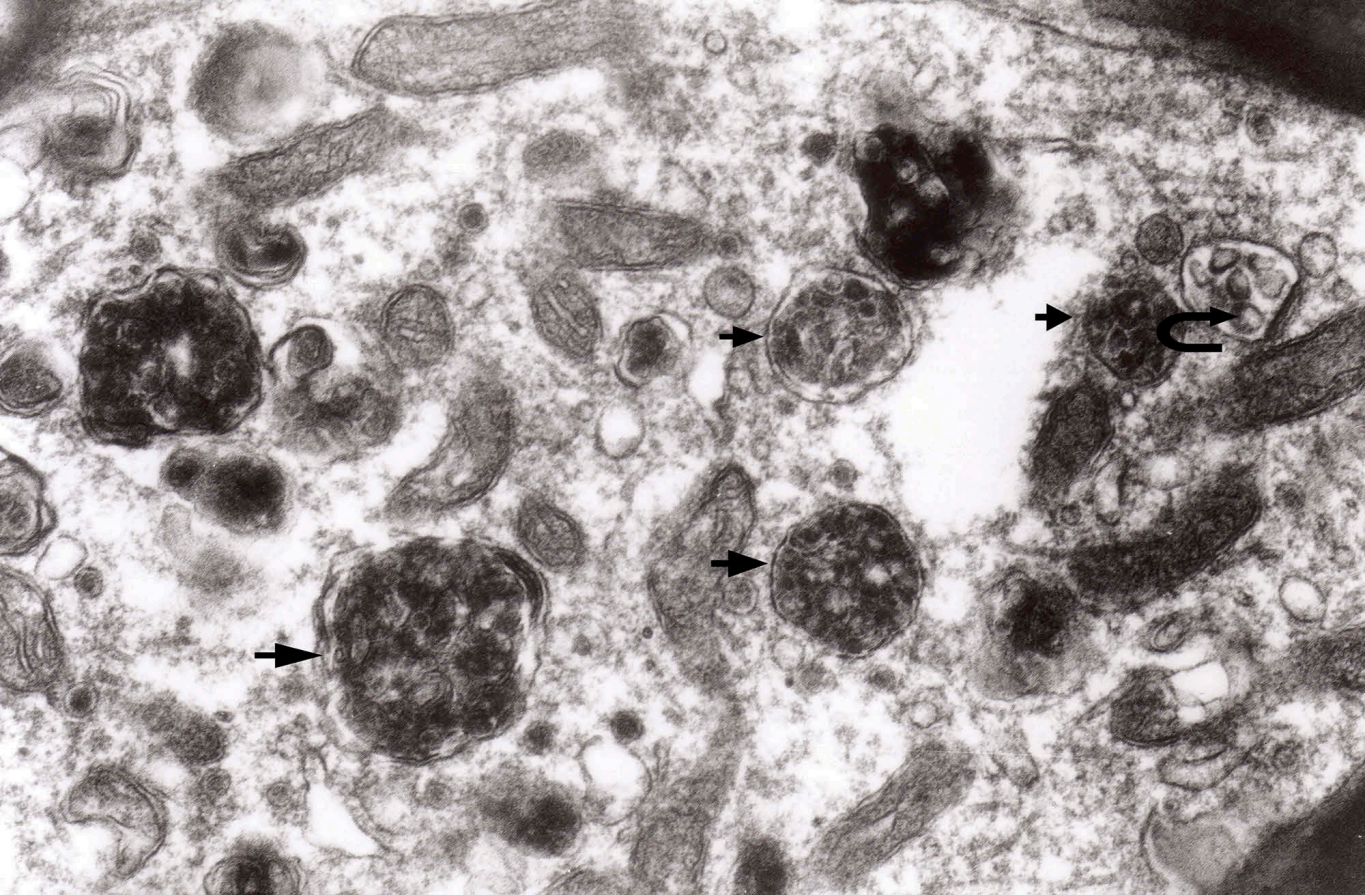
A dystrophic axon with numerous complex MVB (arrows). Those MVB marked with arrows contained vesicles of increased electron density while others (semicircular arrow) contain electron-lucent vesicles; original magnification, x 16,000.

**Figure 18.**
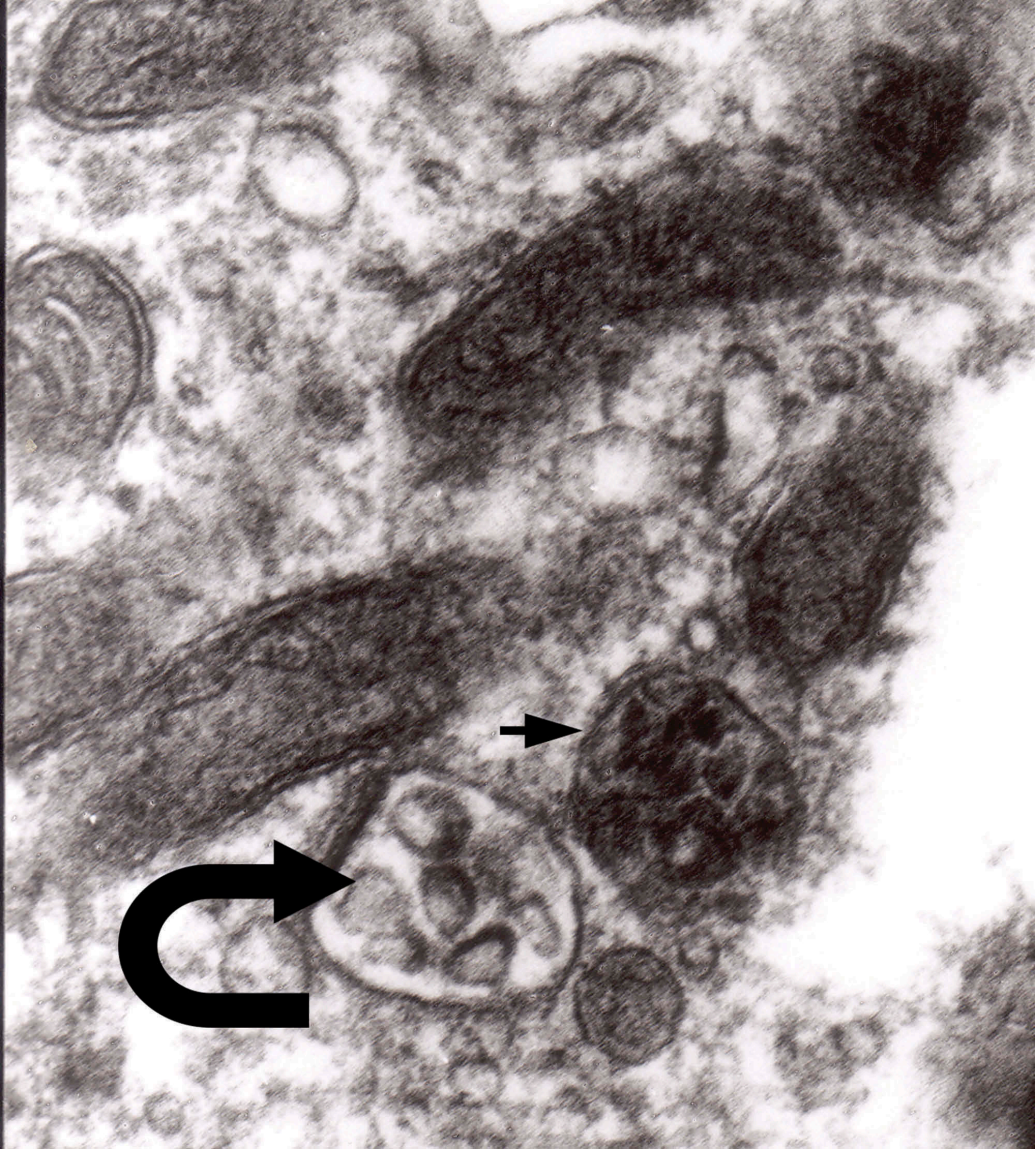
Enlarged fragment of the previous electron-micrograph. Note MVBs – one of normal electron density (semicircular arrow) and one with increased density (arrow); original magnification, x 16,000.

Multivesicular bodies are frequent in both the Matthews’ experiments [46] and in experimental prion diseases described here [28]. In control material they were not very frequent and were of a rather regular form, filled with vesicles with one semicircular end – a ‘cap’ presenting increased electron-density (Figure 16). In prion diseases, MVB accumulated either as round structures (Figures 17–18) or as more elongated forms (Figure 19) [28]. MVBs tend to demonstrate increased density both of inside vesicles and the intravesicular matrix (Figure 17). MVB that accumulate both in constricted sympathetic nerves and in TSEs tend to be numerous, more complex, irregular in contour and somehow flattened (Figure 19).

**Figure 20.**
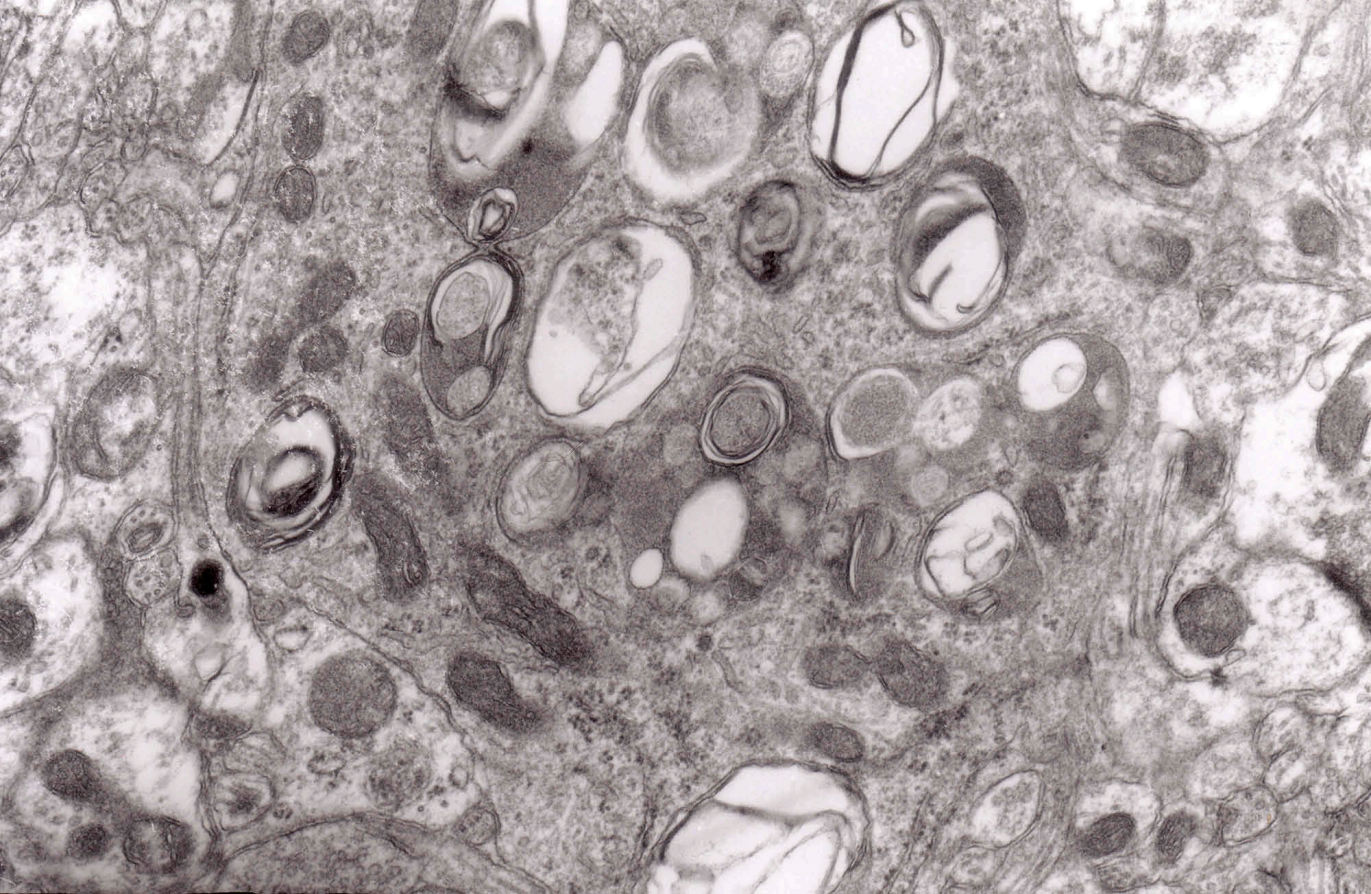
Cytoplasm of the macrophage filled with numerous lysosomes and autophagic vacuoles, original magnification, x 16 000.

In a group of animals 2 to 7 days following ligature [46], late phagosomes were observed in Schwann cells. From obvious reasons, there are no Schwann cells in our TSE model, and Schwann cells function in a similar manner to brain macrophages (Figures 20–21).

With the time between constriction of the nerve and the electron microscopic observation extended to 143 days, the distended axons became less frequent [46].

In this reconstruction, we artificially grouped electron microscopic images according to the framework provided by Matthews in her study of ligatured sympathetic nerves [46]. In prion diseases, those changes were observed simultaneously, but we believe that our isolated images represent a ‘frozen’ processes that affect multiple neurons and their processes at the same time. We could follow distension of axons, accumulations of cellular dense bodies, MVB and autophagic vacuoles as reported by us [28] and by others [47] in experimental scrapie and CJD.

## Neurodegenerative disorders – dystrophic neurites are the major neuronal alterations

The dystrophic neurites in prion diseases are caused by the impairment of axonal transport as was suggested many years ago by Gajdusek [48] and we have shown their presence ultrastructurally in experimental scrapie [39–41], BSE [33], CJD [32,49,50] and GSS [28,51,52] and, finally in chronic wasting disease [53]. Collectively dystrophic neurites form a constant alteration in the prion-affected brains. However, the mechanism(s) of their formation may only be speculated upon. I believe that constriction of neurites, and slowing of the axoplasmic flow leads to their formation.

Using a novel method to study prion diseases, ex-vivo cultures organotypic cerebellar slices, we recapitulated all the scrapie (RML strain) neuropathology at the electron microscopy level [54–56]. The scrapie changes started 5 weeks following inoculation and numerous dystrophic neuritis were readily observed.

One of the most studied prionoid diseases is Alzheimer disease where NAD forms the major part of pathology [7,57,58]. The first description of DN in Alzheimer disease was provided by Lampert [59] – and this definition became the basis of further research ever since. As new transgenic models of AD became available, neuroaxonal dystrophy may be studied in those experimental conditions [60–63]. DN are very plastic, long living and change the size and shape over time; this finding may suggest that what we observe in prion diseases are just different stages of neuritic degeneration. DN are removed by microglial and astrocytic cells [64]. However, the presence of DN within reactive astrocytes were not observed by us in any natural and experimental situations. The exact role of DN in the pathogenesis of Alzheimer disease is unclear; however, they may contribute to the loss of components of the synaptic transmission [62].

Taken together, DN in Alzheimer disease are very similar to those seen in prion diseases. What is their exact role in pathogenesis, remains a mystery.
